# Exploring the role and inter-relationship among nitric oxide, opioids, and K_ATP_ channels in the signaling pathway underlying remote ischemic preconditioning induced cardioprotection in rats

**DOI:** 10.22038/ijbms.2019.34609.8211

**Published:** 2019-07

**Authors:** Sapna Aggarwal, Jasleen Kaur Virdi, Nirmal Singh, Amteshwar Singh Jaggi

**Affiliations:** 1 Department of Pharmaceutical Sciences and Drug Research, Punjabi University Patiala, 147002 India

**Keywords:** Cardioprotection, KATP channels, Nitric oxide, Opioids, Remote ischemic-preconditioning

## Abstract

**Objective(s):**

This study explored the inter-relationship among nitric oxide, opioids, and KATP channels in the signaling pathway underlying remote ischemic preconditioning (RIPC) conferred cardioprotection.

**Materials and Methods:**

Blood pressure cuff was placed around the hind limb of the animal and RIPC was performed by 4 cycles of inflation (5 min) followed by deflation (5 min). An ex vivo Langendorff’s isolated rat heart model was used to induce ischemia (of 30 min duration)-reperfusion (of 120 min duration) injury.

**Results:**

RIPC significantly decreased ischemia-reperfusion associated injury assessed by decrease in myocardial infarct, LDH and CK release, improvement in postischemic left ventricular function, LVDP, dp/dt_max_, and dp/dt_min_. Pretreatment with L-NAME and naloxone abolished RIPC-induced cardioprotection. Moreover, preconditioning with sodium nitroprusside (SNP) and morphine produced a cardioprotective effect in a similar manner to RIPC. L-NAME, but not naloxone, attenuated RIPC and SNP preconditioning-induced increase in serum nitrite levels. Morphine preconditioning did not increase the NO levels, probably suggesting that opioids may be the downstream mediators of NO. Furthermore, glibenclamide and naloxone blocked cardioprotection conferred by morphine and SNP, respectively.

**Conclusion:**

It may be proposed that the actions of NO, opioids, and KATP channels are interlinked. It is possible to suggest that RIPC may induce the release of NO from endothelium, which may trigger the synthesis of endogenous opioids, which in turn may activate heart localized K_ATP_ channels to induce cardioprotection.

## Introduction

Acute myocardial infarction (AMI) remains a leading cause of mortality and morbidity worldwide. Therefore, reperfusion therapeutic strategies including primary percutaneous coronary interventions and fibrinolytic therapy are the mainstay interventions to restore blood flow in ischemic myocardium (1). However, improvement in clinical outcomes after AMI is unsatisfactory due to additional injury conferred by reperfusion itself. Thus, studies have aimed at the adaptive mechanism of the myocardium to make it more resistant against ischemia and to recover its viability on reperfusion. Ischemic preconditioning (IPC) is an adaptive phenomenon in which transient ischemia applied in the vascular territory, delivers protection from deleterious effects of sustained ischemia and delays the myocardial cell death (2). Interestingly, repeated short episodes of ischemia with intermittent reperfusion in different arteries also remotely protect the heart from sustained ischemic injury (3-7) and this phenomenon is termed as remote ischemic preconditioning, which decreases infarction, arrhythmias, and improves post-ischemic contractile function as described by Przyklenk *et al* (8). In the clinical setting, RIPC is shown to attenuate ischemic injury in patients undergoing different forms of cardiac surgery (9-12).

Nitric oxide is an endothelium-derived relaxing factor that is synthesized and released from the endothelium. The endothelium is the chief source of nitric oxide production as it contains two isoforms of nitric oxide synthase (NOS), including constitutively expressive eNOS and inducible form, iNOS. The third isoform nNOS is localized in the nerve fibers (13). Evidence suggests the increment in nitric oxide production during myocardial ischemia and it has been found as a potential candidate to provide protection against myocardial disease (14, 15). Interestingly, nitric oxide and its donor are used clinically in attenuating ischemic injury to the heart (16). Emerging results have implicated the role of nitric oxide in cardioprotection, both as trigger and mediator of ischemic preconditioning (17) and remote preconditioning (18, 19).

Opioids, in addition to the analgesic action in the central nervous system, have been shown to modulate the heart rate, vascular function, and cardiac inotropic effect in the cardiovascular system (20). Notably, the precursors of endogenous opioids are accumulated in cardiac myocytes and their synthesis and release are amplified after ischemia (21, 22). Several research papers have shown that the endogenous opioids act on the cardiac opioids receptors during acute and delayed ischemic preconditioning to produce preconditioning (23, 24). Moreover, administration of non-peptide opioids has been shown to produce an infarct-sparing effect like IPC and cardioprotective effects have been abolished in the presence of naloxone (25). The role of opioid signaling has also been described in RIPC-induced cardioprotection (26, 27). The ATP-sensitive potassium channels (K_ATP_ channel) were first identified in the sarcolemma of cardiac myocytes described by Noma, who observed that activation of K_ATP _channels occur during decrease in the intracellular ATP concentration (28). In the myocardium, two K_ATP_ channel subtypes are present, one on the sarcolemma (sarc K_ATP_) and another on the inner membrane of the mitochondria (mito K_ATP_) (28, 29). Studies have shown that activation of K_ATP_ channels also contributes to RIPC-induced cardioprotection (3, 30, 31). NO, opioids, and K_ATP_ channel have been observed to contribute separately to RIPC-induced cardioprotection, but their inter-relationship underlying RIPC remains unexplored. Therefore, the present study explored the role and inter-relationship among NO, opioids, and K_ATP _channels. 

## Materials and Methods

***Animals***

Wistar rats (150–220 g) were fed a standard laboratory diet and were kept in the laboratory with natural light/dark cycles. Institutional Animal Ethics Committee approved the experimental protocol (approval no. 107/99/CPCSEA/2016/02) and experiments were conducted as per guidelines of CPCSEA, India.

***Drugs and chemicals***

Sodium nitroprusside (Samarth Life Sciences Pvt. Ltd), L-NAME (Cayman Chemicals), morphine (Rusan Healthcare Pvt. Ltd), and naloxone (Samarth Life Sciences Pvt. Ltd) were utilized. Sodium nitroprusside (5 mg/kg) and morphine (10 mg/kg) were injected intraperitoneally and subcutaneously, respectively. L-NAME (10 mg/kg) and naloxone (1 mg/kg) were given intraperitoneally. 

***Induction of remote ischemic preconditioning (RIPC)***

Thiopental sodium (50 mg/kg, IP) was used to anesthetize the rats and RIPC was performed as previously employed in our laboratory (32-34). A neonatal mammalian blood pressure cuff was tied around the hind limb and four cycles of ischemia and reperfusion, of 5 min each, were given by alternatively inflating (up to 150 mm of Hg) and deflating the cuff.

***Isolated heart preparation for Langendorff’s model of I/R injury and measurement of hemodynamic parameters***

Heparin (500 IU/kg, IP) was given to rats and about 15–20 min later, they were sacriﬁced. Thereafter, the heart was isolated and mounted on Langendorff’s apparatus through the aorta, and the heart was perfused with a physiological solution. The pressure at which perfusion was done was set at 70 mm of Hg, and the coronary flow rate was around 7–8 ml/min. The temperature of the isolated heart was maintained at 37 ^°^C by surrounding the heart with a double-walled jacket. After 10 min of stabilization, the heart was subjected to global ischemia (for 30 min) and reperfusion (for 120 min). The contractility parameters such as LVDP, maximum rate of contraction (dp/dt_max_), and maximum rate of relaxation (dp/dt_min_) were measured by inserting a balloon in the left ventricle, and recording of these parameters was done using a Power Lab data acquisition system (AD Instruments). 

***Infarct size determination***

At the end of reperfusion, hearts were removed and kept in the freezer overnight. Thereafter, the heart was cut into slices and staining was done using 1% 2, 3, 5-triphenyl tetrazolium chloride (TTC) (35). The extent of infarction was measured using the volume and weight method (30).

***Estimation of creatine kinase (CK)***

The release of CK in the coronary effluent was quantified using a commercial kit (Agappe Diagnostics Ltd, Kerala, India).

***Estimation of lactate dehydrogenase (LDH)***

The release of lactate dehydrogenase (LDH) in coronary effluent was quantified using the dinitrophenylhydrazone (DNPH) method (36). 

***Estimation of nitrite***

The nitric oxide was determined in the form of nitrite in the serum by a colorimetric assay (37).

***Experimental protocol***

**Figure 1:**
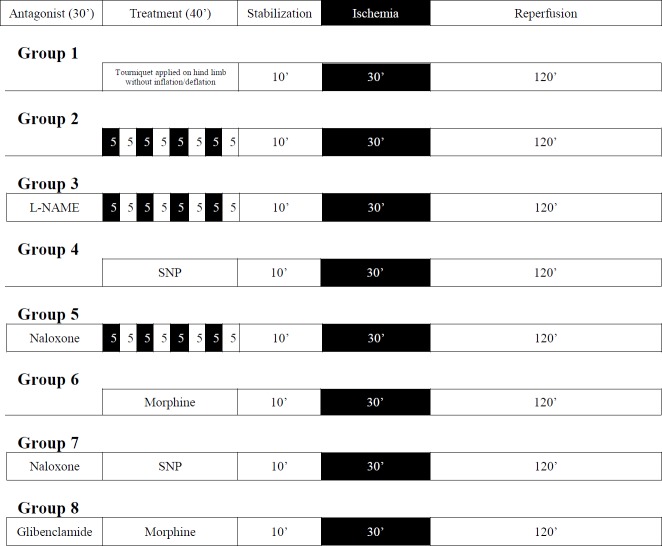
Schematic diagrams of experimental protocol

The rats were randomly distributed into 8 groups, with 6 rats in each group (Figure 1).

**Group I** **(Sham control):** A blood pressure cuff was tied around the hind limb for 40 min, but it was not subjected to inflation/deflation episodes. Thereafter, the animal was sacrificed and the heart was mounted on a Langendorff’s apparatus to provide global ischemia (for 30 min) and reperfusion (for 120 min).

**Group II (RIPC):** A tourniquet was placed around the hind limb and four cycles of ischemia-reperfusion (of 5 min duration each) were induced by inflating and deflating the cuff. Thereafter, the heart was isolated and subjected to ischemia-reperfusion as described in group I. 

**Group III (RIPC + L-NAME):** L-NAME (10 mg/kg, IP) was injected to rats thirty min before RIPC. The rest of the protocol is the same as described in group II. 

**Group IV (NO preconditioning):** The rat was treated with 5 mg/kg of sodium nitroprusside (SNP) intraperitoneally. After 40 min, hearts were subjected to ischemia-reperfusion as per group I. 

**Group V (RIPC + naloxone):** Naloxone (1 mg/kg, IP) was given to rats, thirty min before RIPC. The rest of the protocol is the same as described in group II. 

**Group VI (morphine preconditioning):** The rats were treated with 10 mg/kg of morphine subcutaneously. After 40 min, hearts were subjected to ischemia-reperfusion as per group I.

**Group VII (Naloxone in NO preconditioning): **Naloxone (1 mg/kg, IP) was injected 30 min prior to SNP treatment and the rest of the protocol is the same as described in group IV. 

**Group VIII (Glibenclamide in morphine preconditioning):** Glibenclamide (5 mg/kg, IP) was injected 30 min prior to morphine treatment and the rest of protocol is the same as described in group VI. 

***Statistical analysis***

Graph pad prism 7.00 was used for all statistical analyses. The results were represented as mean±SD. The contractility and biochemical parameters were analyzed using two-way ANOVA followed by Bonferroni’s test. The results of myocardial infarct size and serum nitrite estimations were analyzed using one-way ANOVA followed by Tukey’s test using Graph pad prism 7.00. 

## Results

***Effect of remote preconditioning and different pharmacological intervention on myocardial Infarct size***

**Figure 2:**
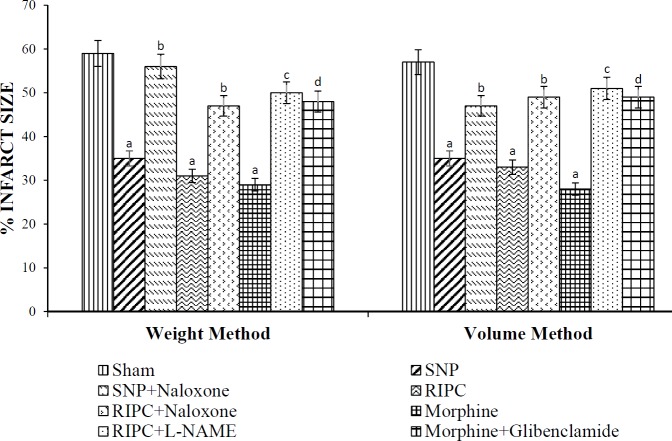
The effect of pharmacological intervention on percentage of myocardial infarct size in rat hearts after 30 min of global ischemia followed by 120 min reperfusion in different experimental groups, determined by the weight and volume method. Values were represented as mean±SD (n=6 in each group). For weight method, F (7, 40)=39.41; for volume method, F (7, 40)=33.21; a=*P<*0.05 vs Sham; b=*P<*0.05 vs RIPC (remote ischemic preconditioning); c=*P<*0.05 vs SNP (sodium nitroprusside); d=*P<*0.05 vs Morphine

In the sham group, approximately 58±4.0 % of myocardium tissue was found to be infarcted following ischemia and reperfusion. However, the myocardial infarction (34±4.0 %) was reduced in RIPC subjected rodents, when compared with the sham group. However, pretreatment with L-NAME and naloxone mitigated RIPC-induced reduction in infarct size. Moreover, exogenous administration of SNP or morphine elicited an infarct-sparing effect, with infarct sizes of 33±3.5 % and 30±4.0 %, respectively. Administration of naloxone in SNP treated and glibenclamide in morphine-treated rats abolished SNP and morphine-induced reduction in myocardial infarction, respectively (Figure 2).

***Effect of RIPC and other interventions on CK release***

**Figure 3:**
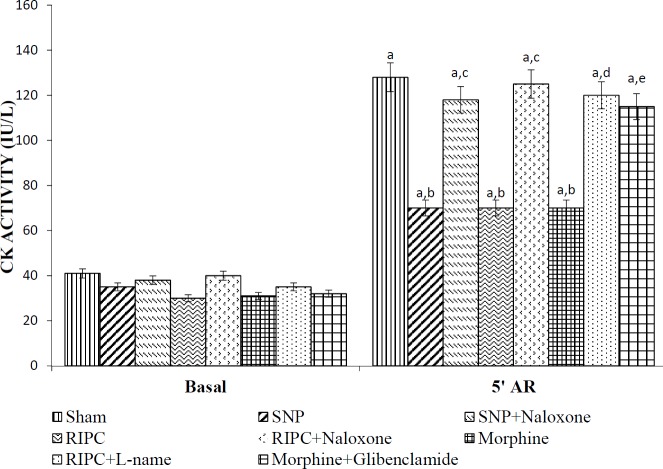
The effect of pharmacological intervention on creatine kinase enzyme activity of rat hearts subjected to 30 min of global ischemia followed by 120 min reperfusion in the coronary effluent of different experimental groups measured at the end of the 10 min stabilization period and 5 min after reperfusion. Values were represented as mean±SD (n=6 in each group). For treatment, F (7, 80)=104.5; for time, F (1, 80)=3920; a=*P<*0.05 vs Basal; b=*P<*0.05 vs Sham; c=*P<*0.05 vs RIPC (Remote ischemic preconditioning); d=*P<*0.05 vs SNP (Sodium nitroprusside); e=*P<*0.05 vs Morphine

RIPC, SNP, and morphine preconditioning significantly abrogated ischemia-reperfusion associated increase in CK release, compared with the sham group. Conversely, pretreatment with L-NAME and naloxone reversed the CK release attenuating effects of RIPC. Further, administration of naloxone and glibenclamide attenuated SNP and morphine-induced decrease in CK levels, respectively (Figure 3).

***Effect of RIPC and other interventions on LDH release***

**Figure 4:**
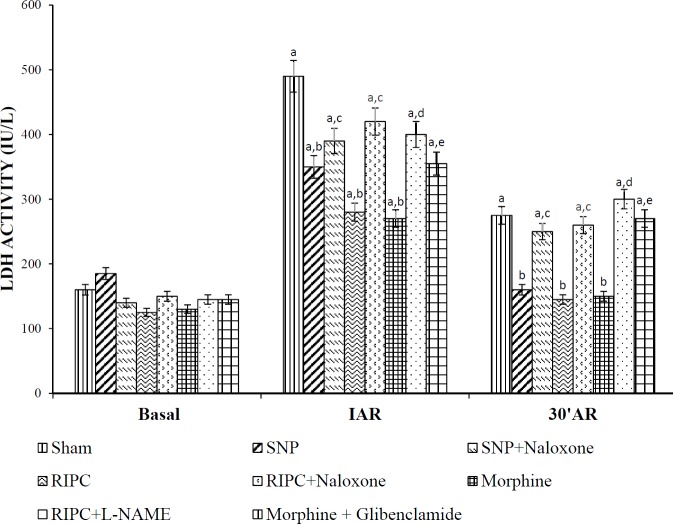
The effect of pharmacological intervention on creatine kinase enzyme activity of rat hearts subjected to 30 min of global ischemia followed by 120 min reperfusion in the coronary effluent of different experimental groups measured at the end of the 10 min stabilization period and 5 min after reperfusion. Values were represented as mean±SD (n=6 in each group). For treatment, F (7, 80)=104.5; for time, F (1, 80)=3920; a=*P<*0.05 vs Basal; b=*P<*0.05 vs Sham; c=*P<*0.05 vs RIPC (Remote ischemic preconditioning); d=*P<*0.05 vs SNP (Sodium nitroprusside); e=*P<*0.05 vs Morphine

RIPC along with pharmacological preconditioning with SNP and morphine significantly alleviated ischemia-reperfusion associated increase in LDH release at different time intervals. Conversely, pretreatment with L-NAME and naloxone attenuated the LDH attenuating effects of RIPC. Further, LDH release was significantly higher in SNP+naloxone and morphine+glibenclamide groups as compared with SNP and morphine-treated rats, respectively, suggesting that naloxone and glibenclamide attenuated LDH attenuating actions of SNP and morphine respectively (Figure 4).

***Effect of RIPC and other interventions on serum nitrite***

**Figure 5:**
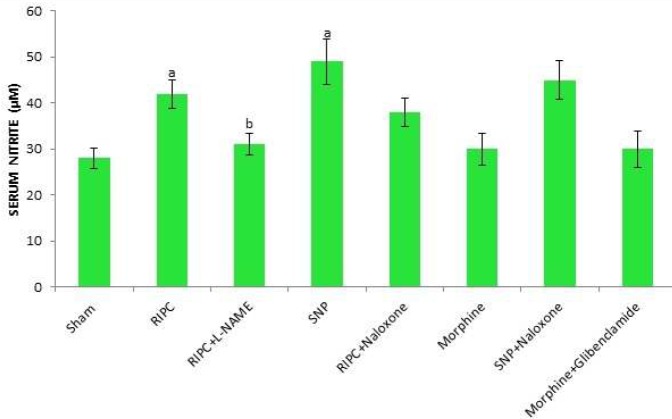
The effect of pharmacological intervention on serum nitrite levels in rats. Values were represented as mean±SD (n=6 in each group). F (7, 40) = 21.34; a =*P<*0.05vs Sham; b =*P<*0.05 vs RIPC (remote ischemic preconditioning)

There was a significant increase in serum nitrite levels following RIPC and SNP preconditioning as compared with the sham group. Pretreatment with L-NAME signiﬁcantly reduced the serum nitrite levels in RIPC subjected rodents. However, no significant difference in the nitrite levels was observed in the presence of naloxone in RIPC and SNP treated animals. In other words, naloxone did not alter the nitrite levels in RIPC and SNP treated rats. Moreover, no changes in serum nitrite levels were observed in morphine and morphine+glibenclamide treated rats (Figure 5).

***Effect of RIPC and other interventions on contractility parameters***

After thirty min of global ischemia, a marked decrease in contractility parameters, ie, LVDP, dp/dt_max_, and -dp/dt_min _was observed in reperfusion. RIPC along with pharmacological preconditioning with SNP and morphine enhanced the contractility parameters of ventricles following global ischemia when compared with the sham group at different time periods of reperfusion. Pretreatment with L-NAME and naloxone attenuated the improvement in postischemic cardiac performance in RIPC treated animals. Moreover, naloxone and glibenclamide also abolished the improvement in contractile function of the left ventricle afforded by SNP and morphine, respectively (Tables 1, 2, and 3).

## Discussion

In the present investigation, global ischemia followed by reperfusion produced marked injury assessed in the form of myocardial infarction; LDH, CK release, and functional parameters, i.e., LVDP, dp/dt_max_, dp/dt_min_. However, RIPC significantly alleviated myocardial injury, which is in consonance with earlier observations of our laboratory (32-34). The hind limb model of RIPC is a widely accepted procedure as it is easier, noninvasive, feasible, and clinically compatible. 

 Another important finding of this study is the up-regulation of nitric oxide levels, measured as increased nitrite levels, in the serum of remote preconditioned animals. However, this effect was blocked by pretreatment with L-NAME (NOS inhibitor), which also alleviated the cardioprotective effects of RIPC. The up-regulation of nitric oxide as a trigger may account for the cardioprotective effects of RIPC, as documented in other previous research studies (38, 18). Furthermore, exogenous administration of NO donor, SNP (pharmacological preconditioning) mimicked RIPC-induced cardioprotection, emphasizing the critical role of nitric oxide in RIPC mediated protective effects. In the present investigation, naloxone abrogated RIPC conferred protective effects suggesting that the opioid receptors are activated during RIPC. This is in consonance with our earlier findings, showing that mesenteric artery occlusion or infrarenal aortic occlusion-induced protection against sustained ischemia is abolished in the presence of naloxone (26, 39). Moreover, pharmacological preconditioning with morphine also mimicked the protective effects of RIPC again emphasizing the critical role of opioids in cardioprotection. 

The inter-relationship between opioids and nitric oxide may be analyzed by studying the changes in nitrite levels in response to different interventions. Despite the increased levels of nitrite in the serum of remote preconditioned animals and SNP administered animals, the protection conferred by RIPC and SNP preconditioning was alleviated with the pretreatment of naloxone. Naloxone non-selectively blocks the action of endogenous opioids (40). Naloxone-induced reduction of NO-dependent cardioprotective effects, without altering nitrite serum levels, which suggests that opioids may be the downstream mediators of nitric oxide. Furthermore, morphine preconditioning produced a cardioprotective effect without increasing nitrite levels in serum suggesting that opioid-induced cardioprotection is not dependent on an increase in NO levels. This argument is supported by a study of Armstead who demonstrated that nitric oxide triggers the release of endogenous opioids under hypoxic condition (41). Therefore, it may be suggested that remote preconditioning induces nitric oxide release, which may produce a cardioprotective effect through endogenous opioids.

There are a number of reports suggesting that K_ATP_ channels play a key role as mediator/ end effector in the signaling pathway underlying RIPC against sustained ischemia (42, 43). Our previous study also pointed to the important role of the K_ATP_ channel in cardioprotection elicited by RIPC (30). Additionally, the results of the present study showed that blockade of K_ATP_ channels with glibenclamide (non-specific K_ATP_ channels antagonist) reversed the cardioprotective effect of morphine preconditioned animals. These findings are in line with the study of Liang and Gross, which demonstrated that morphine produces preconditioning-like effects via K_ATP_ channels dependent mechanism in cardiac myocytes of intact heart (44). Therefore, it may be possible to suggest that opioid produces cardioprotection through K_ATP_ channel opening.

Based on these, it may be proposed that the actions of NO, opioids, and K_ATP_ channels are interlinked. It is possible to suggest that RIPC may induce the release of NO from endothelium, which may trigger the synthesis of endogenous opioids, which in turn may activate heart localized K_ATP_ channels to induce cardioprotection. 

**Table 1:** The effect of pharmacological intervention on post ischemic left ventricular developed pressure (LVDP) in rat hearts subjected to 30 min global ischemia followed by 120 min reperfusion at different time intervals. All values were represented as absolute values of LVDP in mmHg. Values were represented as mean±SD (n=6 in each group). For treatment, F (7, 200)=50.65; for time, F (4, 200)=587.9; a =*P<*0.05 vs Basal; b =*P<*0.05 vs Sham; c =*P<*0.05 vs RIPC (remote ischemic preconditioning); d =*P<*0.05 vs SNP (sodium nitroprusside); e =*P<*0.05 vs morphine

**Group**	**Baseline**	**Reperfusion**			
		**5 min**	**30 min**	**60 min**	**120 min**
**Sham**	93±8	48±4^a^	54±4.0^a^	42±3^a^	26±3^a^
**RIPC**	98±10	65±5^a,b^	78±6^b^	53±3^a^	43±4^a,b^
**RIPC+L-NAME**	96±9	45±4^a,c^	57±4^a,c^	41±3^a^	32±2^a^
**SNP**	106±14	72±6^a,b^	84±7^a,b^	64±4^a,b^	57±4^a,b^
**RIPC+Naloxone**	94±10	43±6^a,c^	60±5^a,c^	39±2^a^	36±4^a^
**Morphine**	108±16	73±7^a,b^	83±5^a,b^	58±4^a^	51±6^a,b^
**SNP+Naloxone**	104±11	53±6^a,d^	66±6^a,d^	43±5^a,d^	30±3^a,d^
**Morphine+** **Glibenclamide**	106±13	56±6^a,e^	65±5^a,e^	49±6^a^	38±5^a,e^

**Table 2:** The effect of pharmacological intervention on post ischemic maximum rate of contraction (dp/dtmax) in rat hearts subjected to 30 min global ischemia followed by 120 min reperfusion at different time intervals. All values are represented as absolute values of dp/dtmax in mmHg/sec. Values were represented as mean±SD (n=6 in each group). For treatment, F (7, 200)=63.9; for time, F (4, 200)=1287; a=*P<*0.05 vs Basal; b =*P<*0.05 vs Sham; c =*P<*0.05 vs RIPC (remote ischemic preconditioning); d =*P<*0.05 vs SNP (sodium nitroprusside); e=*P<*0.05 vs morphine

**Group**	**Baseline**	**Reperfusion**			
		**5 min**	**30 min**	**60 min**	**120 min**
**Sham**	3325±259	1448±129^a^	1857±153^a^	1280±123^a^	951±102^a^
**RIPC**	3548±271	2035±210^a,b^	2292±250^a,b^	1828±149^a,b^	1477±120^a,b^
**RIPC+L-NAME**	3465±206	1539±165^a,c^	1720±166^a,c^	1236±135^a,c^	1078±112^a^
**SNP**	3626±285	2115±218^a,b^	2487±130^a,b^	1980±152^a,b^	1496±156^a,b^
**RIPC+Naloxone**	3298±228	1495±123^a,b^	1802±142^a,b^	1174±104^a,c^	1004±100^a,c^
**Morphine**	3685±295	2245±1471^a,b^	2518±189^a,b^	1938±139^a,b^	1366±120^a,b^
**SNP+Naloxone**	3585±247	1588±130^a,d^	1890±119^a,d^	1368±132^a,d^	984±90^a,d^
**Morphine+** **Glibenclamide**	3695±268	1621±124^a,e^	1960±134^a,e^	1681±169^a^	1034±97^a^

**Table 3:** The effect of pharmacological intervention on post ischemic maximum rate of relaxation (-dp/dtmin) in rat hearts subjected to 30 min global ischemia followed by 120 min reperfusion at different time intervals. All values are represented as absolute values of dp/dtmin in mmHg/sec. Values were represented as mean ± SD (n=6 in each group). For treatment, F (7, 200) =63.9; for time, F (4, 200) =1287; a =*P*<0.05 vs Basal; b =*P<*0.05 vs Sham; c =*P<*0.05 vs RIPC (remote ischemic preconditioning); d =*P<*0.05 vs SNP (sodium nitroprusside); e =*P<*0.05 vs morphine

**Group**	**Baseline**	**Reperfusion**			
		**5 min**	**30 min**	**60 min**	**120 min**
**Sham**	2947±251	1234±129^a^	1438±15^a^	1139±132^a^	952±98^a^
**RIPC**	3227±258	1910±195^a,b^	2061±192^a,b^	1640±172^a,b^	1383±15^a,b^
**RIPC+L-NAME**	3071±228	1342±158^a,c^	1479±123^a^	1176±109^a,c^	988±87^a^
**SNP**	3296±272	2034±216^a,b^	2249±228^a,b^	1761±168^a,b^	1442±166^a,b^
**RIPC+Naloxone**	3119±243	1398±125^a,c^	1505±129^a^	1196±106^a,c^	995±95^a^
**Morphine**	3340±290	2110±22^a,b^	2289±205^a,b^	1812±183^a,b^	1558±179^a,b^
**SNP+Naloxone**	3186±263	1439±143^a,d^	1687±182^a,d^	1249±139^a,d^	965±92^a,d^
**Morphine+** **Glibenclamide**	3214±259	1562±159^a,e^	1736±178^a,e^	1499±145^a^	1164±125^a,e^

## Conclusion

It may be concluded that nitric oxide is an upstream mediator of opioids, which then subsequently activates the K_ATP_ channel to produce cardioprotection during remote preconditioning. 
